# Eu^3+^ and Tb^3+^ coordination compounds with phenyl-containing carbacylamidophosphates: comparison with selected Ln^3+^ β-diketonates

**DOI:** 10.3389/fchem.2023.1188314

**Published:** 2023-05-15

**Authors:** Nataliia S. Kariaka, Aneta Lipa, Albano N. Carneiro Neto, Oscar L. Malta, Paula Gawryszewska, Volodymyr M. Amirkhanov

**Affiliations:** ^1^ Inorganic Chemistry Department, Taras Shevchenko National University of Kyiv, Kyiv, Ukraine; ^2^ Faculty of Chemistry, University of Wroclaw, Wroclaw, Poland; ^3^ Physics Department, CICECO-Aveiro Institute of Materials, University of Aveiro, Aveiro, Portugal; ^4^ Departamento de Química Fundamental, Universidade Federal de Pernambuco, Recife, Brazil

**Keywords:** lanthanide, luminescence, carbacylamidophosphates, β-diketone, thermal gravimetric analysis, photosensitisation, energy transfer

## Abstract

Materials based on Eu^3+^ and Tb^3+^ coordination compounds are of great interest due to their strong red and green luminescence. Appropriate selection of ligands plays a huge role in optimizing their photophysical properties. Another very helpful instrument for such optimization is theoretical modelling, which permits the prediction of the emissive properties of materials through intramolecular energy transfer analysis. The ligands that allow for achieving high efficiency of Eu^3+^ and Tb^3+^ emissions include carbacylamidophosphates (CAPh, HL). In this brief review, we summarize recent research for lanthanides CAPh-based coordination compounds of general formulas Cat[LnL]_4_, [LnL_3_Q] and [Ln(HL)_3_(NO_3_)_3_], where Cat^+^ = Cs^+^, NEt4^+^, PPh_4_^+^ and Q = 1,10-phenanthroline, 2,2-bipyridine or triphenylphosphine oxide, involving the use of thermal gravimetric analysis, X-ray analysis, and absorption and luminescence spectroscopy. We carried out a comparison with selected Ln^3+^ β-diketonates. Possibilities and developments of theoretical calculations on energy transfer rates are also presented.

## 1 Introduction

During recent years, coordination compounds of lanthanides have been the focus of attention of many research groups around the world because of their practical application in modern technologies. Special attention is paid to research aimed at the search for luminescent materials. From this point of view, lanthanide coordination compounds attract attention primarily due to the specificity of the luminescence mechanism caused by f-f transitions, which allows for obtaining monochromatic radiation, which is uncharacteristic for phosphors of a purely organic nature. The luminescence of triply charged lanthanide ions (Ln^3+^) is the subject of research in many scientific fields from laser physics to molecular biology, which is due to interest not only from a fundamental point of view but also from the possibility of practical application in light generators, sensors, optical amplifiers, lasers, flat displays, fluorescent lighting, and medical diagnostics (Bünzli, 2017).

The problem of the low absorption capacity of 4f-4f transitions of Ln^3+^ (Bünzli, 2015) in coordination compounds is solved by the creation and selection of organic ligands capable of sensitizing the emission of lanthanides by transferring the energy absorbed by them to the metal, as well as by partially removing the parity ban on transitions inside the f-shell. Therefore, the search for new ligands, lanthanide emission sensitizers, and the synthesis and research of compounds based on them is a current task of modern coordination chemistry.

Chemists are constructing a great variety of new molecules that can be combined with lanthanide ions to give efficient lanthanide luminescence. A significant number of successful studies of luminescent lanthanides coordination compounds has been found among coordination compounds based on β-diketonate ligands. These compounds are the most popular and the most intensively investigated rare-earth coordination compounds partially because many different β-diketones are commercially available and the fact that the synthesis of the corresponding rare-earth coordination compounds is relatively easy. Other new promising ligands to design highly luminescent materials for practical use are carbacylamidophosphates (CAPhs), which can be considered as P,N-substituted analogues of β-diketones containing a chelating core C(O)N(H)P(O) (Amirkhanov et al., 2014). The phosphoryl group, present in these ligands, possess high affinity to lanthanides. Moreover, if compared with well-known β-diketones antennas, CAPhs contain up to three highly absorbing groups, and the high energy vibrations in the chelating fragment of CAPhs are reduced by replacing C with N and one C=O group with P=O.

In recent years, the field of lanthanide-based materials has significantly advanced with the introduction of various theoretical approaches and methods (Kushida, 1973; Malta et al., 1998; Maron and Eisenstein, 2001; Malta, 2008; Dutra et al., 2014; Wen et al., 2014; Tanner et al., 2018; Georgieva et al., 2020; Suta and Meijerink, 2020; Carneiro Neto et al., 2021; Moura et al., 2021a; Carneiro Neto et al., 2022; Moura et al., 2022a; Salerno et al., 2022). These procedures have proven successful in predicting and explaining the photophysical properties of these materials. For instance, the combination of density functional theory (DFT), intramolecular energy transfer (IET) theory, and rate equation modelling has enabled researchers to calculate the quantum yield, which is the ratio between absorbed and emitted photons (Kasprzycka et al., 2020; Carneiro Neto et al., 2022; Moura et al., 2022b; Moura et al., 2022c). These theoretical advancements have allowed for the design of lanthanide-based materials with tailored photophysical properties suitable for a wide range of applications, such as optical sensing (Almeida et al., 2022; Ramalho et al., 2022), optical thermometry (Lyubov et al., 2022; Salerno et al., 2022), bioimaging (Piñol et al., 2020; Yao et al., 2023), and light-emitting devices (Kido et al., 1993; Hernández‐Rodríguez et al., 2023).

This review is about recent investigations on the luminescence of carbacylamidophosphates (CAPh) based on Eu^3+^ and Tb^3+^ coordination compounds. Herein we will focus on the Ln^3+^ coordination compounds based on phenyl-containing CAPhs, and on the features of their luminescence compared to Eu^3+^ and Tb^3+^ β-diketonates.

## 2 A brief history and main concepts of lanthanide photophysics

The unusually sharp absorption lines of rare earth compounds were first observed by Becquerel in 1906 when he measured the spectrum of the mineral xenotime (YPO_4_ containing traces of Er, Ce, and Th) (Becquerel, 1906). Around 1930, it was suggested that those lines could be due to electronic transitions within the Ln^3+^ 4f configuration - f-f transitions (Becquerel, 1929; Bethe, 1930). Thus, the key to understanding the spectral properties of lanthanides is their specific electronic configuration: the ground state of Ln^3+^ ions [Xe]4f^*n*^ (*n* = 0–14) is energetically well separated from the [Xe]4f^*n−1*^5d^1^ configuration (∆Е >32,000 cm^−1^). In addition, there is the shielding of the 4f-orbitals by the “xenon core” (54 electrons), in particular, by the 5s^2^ 5p^6^ sublevels, which makes the valence 4f-orbitals “internal”. Therefore, electrons in the 4f shell do not play a role in the chemical bonding between the lanthanide ions and the ligands. As a consequence, the influence of the lanthanide coordination environment on the optical transitions within the 4f shell is small, resulting in sharp-line spectra.

If these transitions are indeed intraconfigurational, the question remained as to how these transitions acquire their strength: formally they are forbidden by the Laporte (or parity) selection rule. In 1937, van Vleck addressed this puzzle and showed that they become partially allowed as electronic dipole transitions by an admixture of configurations of opposite parity, such as the 4f^*n−1*^5d^1^ configuration (Van Vleck, 1937). Some 4f-4f transitions are allowed as magnetic dipole transitions, and both schemes yield oscillator strengths of the same order of magnitude.

**FIGURE 1:**
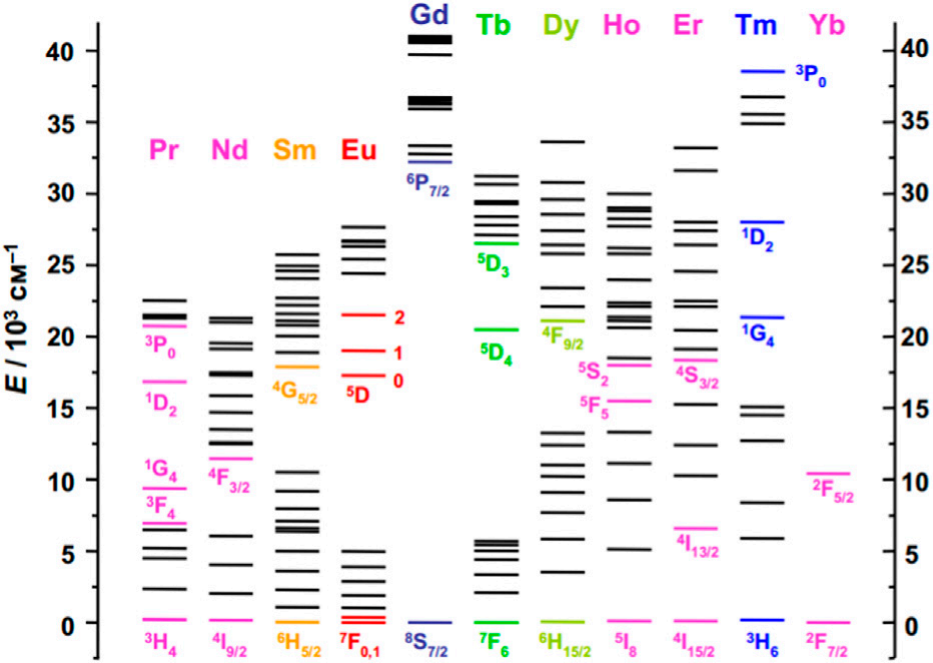
Energy level diagram for some Ln^3+^ ions (Dieke, 1968).

Dieke’s diagram (Figure 1) provides a comprehensive overview of the energy levels of the lanthanide series (Carnall et al., 1968a; Carnall et al., 1968b; Carnall et al., 1968c; Carnall et al., 1968d; Dieke, 1968; Carnall et al., 1979). It has been demonstrated that the position of Ln^3+^ energy levels is relatively insensitive to the chemical environment (Carnall et al., 1989; Peijzel et al., 2005). Therefore, this diagram can be applied to describe the energy levels of lanthanide ions in different compounds. However, changes in the intensity and width of spectral lines may occur depending on the local environment of the lanthanide ions (Judd, 1962; Ofelt, 1962; Mason et al., 1974; Judd, 1979; Malta, 1982; Moura et al., 2016). This highlights the importance of the local environment’s effects on the lanthanide ions, even though the energy level remains unchanged.

Most lanthanide ions are known to exhibit luminescent properties, but their intensity varies depending on the type of Ln^3+^. It is important to note that La^3+^ lacks 4f-electrons while Lu^3+^ has a filled 4f-shell, meaning that these ions do not exhibit 4f luminescence. However, the remaining lanthanides cover the entire spectral range from ultraviolet (Gd^3+^), visible light (Sm^3+^ - orange; Eu^3+^, Er^3+^ - red; Tb^3+^, Er^3+^ - green; Dy^3+^ - yellow; Tm^3+^ - blue) and near-infrared (Pr^3+^, Nd^3+^, Ho^3+^, Er^3+^, and Yb^3+^).

In 1942, it was discovered that certain organic ligands can enhance the luminescence of lanthanides through sensitization (Weissman, 1942; Yuster and Weissman, 1949). In the 1960s, systematic studies on luminescent lanthanide coordination compounds and their photophysics began to emerge (Crosby and Kasha, 1958; Crosby et al., 1961; Crosby et al., 1962; Bauer et al., 1964; Bhaumik, 1964; Melby et al., 1964; Bhaumik and El-Sayed, 1965). These studies mainly focused on Eu^3+^ and Tb^3+^ β-diketonates, and many of the photophysical concepts discovered at that time are still used today. Lanthanide β-diketonates remain among the most studied luminescent compounds due to their commercial availability, easy synthesis of the coordination compounds, and excellent luminescent properties.

In 1997, Malta introduced the formalism of the IET theory (Malta, 1997). This theory allowed for the calculation of energy transfer rates from organic excited states (*e.g.*, S_1_ and T_1_) to the 4f excited states of Ln^3+^ using multipolar mechanisms such as dipole-dipole and dipole-quadrupole interactions. This was a significant advancement in the understanding of energy transfer mechanisms involving lanthanide ions. Later on, Gonçalves e Silva and Malta added an expression for the exchange mechanism (e Silva and Malta, 1997; Malta and Gonçalves e Silva, 1998), which further expanded the understanding of the energy transfer process. The addition of the exchange mechanism allowed for a more comprehensive understanding of the complex IET process involved in lanthanide-based compounds.

These works were particularly important because they filled a gap of 55 years in the quantitative approach to the IET theory since Weissman’s work in 1942 (Weissman, 1942). With these new developments, researchers had a more complete understanding of energy transfer mechanisms and could more accurately predict the rates of energy transfer between organic excited states and the 4f excited states of lanthanide ions. In recent years, modern computational programs such as LUMPAC (Dutra et al., 2014) and JOYSpectra (Moura et al., 2021a) have been developed and implemented with the IET equations to aid the lanthanide spectroscopy community in understanding the underlying photophysical processes of lanthanide-based compounds. These programs have greatly benefited researchers in predicting and understanding the complex energy transfer mechanisms that occur in lanthanide photophysics.

## 3 Sensitization, deactivation of lanthanide luminescence, and strategies to enhance the efficiency of Ln^3+^ luminescence in coordination compounds

**FIGURE 2:**
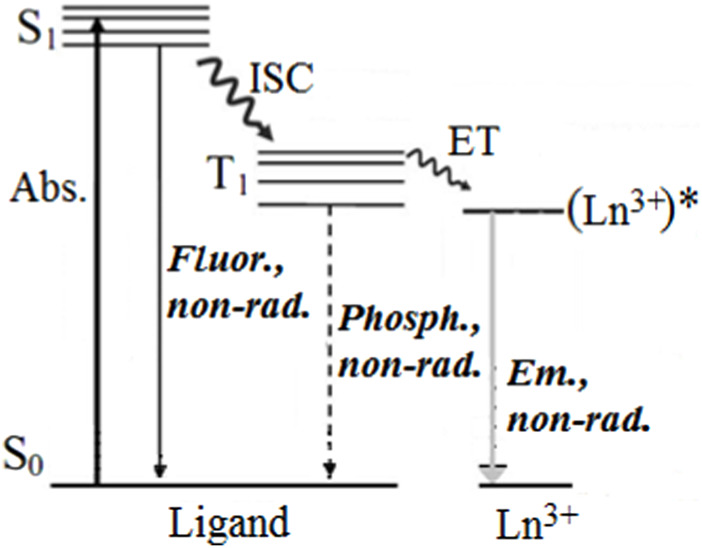
Simplified energy level diagram of the Ln^3+^ sensitization.

Lanthanide ions have small 4f-4f absorption coefficients (Bünzli, 2015), making them difficult to excite directly. As a result, an indirect excitation approach known as sensitization or the “antenna effect” is commonly used in practical applications. In coordination compounds, a ligand is used as a sensitizer for lanthanide ion luminescence and also serves to protect the Ln^3+^ from solvent or other molecules, thus preventing nonradiative relaxations. As previously mentioned, the sensitization of lanthanides in coordination compounds was observed and proposed for the first time by Weissman (Yuster and Weissman, 1949), who suggested that a photon can be absorbed by an organic ligand with high absorption cross-section and transferred by the IET to the resonant levels of the Ln^3+^. Crosby and colleagues published the complete mechanism, introducing the role of the ligand triplet state in the IET process (Crosby et al., 1961; Crosby et al., 1962). Figure 2 shows a simplified Jablonski energy level scheme of the IET sensitization process.

The sensitization of the Ln^3+^ emission (Em.) occurs through several stages, including absorption of light by the ligand (Abs.), intersystem crossing (ISC), and intramolecular energy transfer (ligand-to-Ln^3+^). Additionally, direct energy transfer from the ligand’s excited singlet state S_1_ to the lanthanide can occur (Kasprzycka et al., 2017; Aquino et al., 2021; Moura et al., 2021b; Tanner et al., 2022). The excitation energy can be partially lost due to fluorescence emission (S_1_→S_0_), phosphorescence emission (T_1_→S_0_), and non-radiative relaxations in the middle of the sensitization process. Therefore, the efficiency of sensitization (η_sens_) and the Ln^3+^ emission greatly affect the overall emission quantum yield ( $Q_{\mathrm{Ln}}^{L}$ ):

$$Q_{\mathrm{Ln}}^{L}=\eta_{sens}∙Q_{\mathrm{Ln}}^{\mathrm{Ln}}$$ (1)

The influence of the first component (η_sens_) is regulated most often by selecting ligands with the optimal value of the singlet and triplet levels positions. The latter can be determined by the low-temperature phosphorescence spectra of La^3+^, Gd^3+^, and Lu^3+^ coordination compounds (Brito et al., 2010; Carneiro Neto et al., 2019). The optimal ranges of the energy gap between the ligand’s lowest triplet state and the emissive level of the lanthanide (ΔЕ) have been estimated as 2,500–3,500 cm^−1^ for an efficient ligand-to-metal energy transfer (Bünzli et al., 2010). When the energy gap is less than ∼1900 cm^−1^, the backward IET may take place (Sato and Wada, 1970), leading to a decrease in luminescence efficiency. On the other hand, if ΔЕ is too high, it increases the probability of non-radiative decay, making it difficult to efficiently transfer energy to the Ln^3+^. Although this rule is generally used in the design of efficient lanthanide-based phosphors, it does not always work in practice due to the more complicated nature of the IET process. Factors such as the influence of charge transfer states (Gérard et al., 1994; Petoud et al., 1999; Faustino et al., 2005; Kasprzycka et al., 2020; Kasprzycka et al., 2022), participation of several IET pathways between the ligand and the Ln^3+^ ion (Moura et al., 2021a; Moura et al., 2021b), donor-acceptor distances (Carneiro Neto et al., 2019; Kasprzycka et al., 2020; Moura et al., 2022c), and temperature (Carneiro Neto et al., 2021; Salerno et al., 2022) can also have an impact on the photophysical process.

The intrinsic quantum yield $Q_{\mathrm{Ln}}^{\mathrm{Ln}}$ is a ratio that compares the radiative rate to the total decay rate (radiative and non-radiative) of an emitting level. Non-radiative processes are influenced by quenching groups, such as O-H, N-H, and C-H, that are present in or near the coordination sphere (Heller, 1966; Haas and Stein, 1971). To reduce non-radiative transitions, solvents should be removed from the lanthanide coordination sphere, and ligands without quenching groups can be used in chelating cores. Mixed ligand complexation can be used to achieve this goal (Gawryszewska et al., 2005). In addition, non-aqueous solvents or deuterated molecules of organic solvents can also be applied in the synthesis of coordination compounds (Piguet and Bünzli, 1999).

Malta’s theory is used to describe the IET mechanisms between the donor (ligand) and acceptor (Ln^3+^) in the presence of a perturbation. This theory builds upon Fermi’s golden rule, which describes for a system the probability of making a transition from an initial state to a final state due to a perturbation. In this framework, three interactions or mechanisms are introduced: the dipole-dipole ( $W_{d-d}$ ), dipole-multipole ( $W_{d-m}$ ), and exchange ( $W_{ex}$ ) mechanisms (Malta, 1997; Malta and Gonçalves e Silva, 1998; Carneiro Neto et al., 2019),

$$W_{d-d}=\frac{2\pi }{\hbar }\left(\frac{S_{d}^{L}∙S_{d}^{\mathrm{Ln}}}{G∙R_{L}^{6}}\right)F$$ (2)

$$W_{d-m}=\frac{2\pi }{\hbar }\left[\frac{S_{d}^{L}}{G}\left(\underset{\lambda }{\sum }\frac{S_{\lambda }^{\mathrm{Ln}}}{{\left(R_{L}^{\lambda +2}\right)}^{2}}\right)\right]F$$ (3)

$$W_{ex}=\frac{2\pi }{\hbar }\left(\frac{S_{ex}^{L}∙S_{ex}^{\mathrm{Ln}}}{{G∙R}_{L}^{4}}\right)F$$ (4)

where,

$$S_{d}^{\mathrm{Ln}}=\frac{2e^{2}{\left(1-\sigma_{1}\right)}^{2}}{\left(2J+1\right)}\underset{\lambda }{\sum }\Omega_{\lambda }^{FED}{\langle \psi^{*}J^{*}\left\|U^{\left(\lambda \right)}\right\|\psi J\rangle }^{2}$$ (5)

$$S_{\lambda }^{\mathrm{Ln}}=\frac{e^{2}{\left(1-\sigma_{\lambda }\right)}^{2}\left(\lambda +1\right)}{\left(2J+1\right)}{\langle r^{\lambda }\rangle }^{2}{\langle f\left\|C^{\left(\lambda \right)}\right\|f\rangle }^{2}{\langle \psi^{*}J^{*}\left\|U^{\left(\lambda \right)}\right\|\psi J\rangle }^{2}$$ (6)

$$S_{ex}^{L}=\frac{{4\left(1-\sigma_{0}\right)}^{2}}{3\left(2J+1\right)}e^{2}{\langle \psi^{*}J^{*}\left\|S\right\|\psi J\rangle }^{2}$$ (7)

$$S_{ex}^{\mathrm{Ln}}=e^{2}\underset{m}{\sum }{|\langle \phi |\underset{j}{\sum }\mu_{z}\left(j\right)s_{m}\left(j\right)|\phi^{*}\rangle |}^{2}$$ (8)

Equations 2–4 provide key factors for understanding the interaction between the donor and acceptor in an energy transfer process. $R_{L}$ represents the distance between the donor and acceptor, F is the spectral overlap factor that considers the energy mismatch between the donor and acceptor transitions, and G is the donor state degeneracy, which depends on whether the donor is in a singlet or triplet state. Note the similarities of these equations with Fermi’s golden rule $W_{i\to k}=\left(2\pi /\hbar \right){\left|\langle i\left|V\right|k\rangle \right|}^{2}\rho$ , which provides the rate of transition between two states ( $\left|i\right.\rangle$ and $\left|k\right.\rangle$ ) based on their interaction strength. Additionally, from the Ln^3+^ side, Eq. 5 ( $S_{d}^{\mathrm{Ln}}$ ) represents the dipole strength, while Eq. 6 ( $S_{\lambda }^{\mathrm{Ln}}$ ) represents the strength of the multipole expansion. From the Ligand side, $S_{d}^{L}$ represents the dipole strength of the donor transition involved in the energy transfer process (S_1_→S_0_ or T_1_→S_0_), which can be estimated using spin-orbit coupling calculations (Carneiro Neto et al., 2022). These calculations typically lead to values in the order of $S_{d}^{L}∼10^{-37}$ esu^2^‧cm^2^ for S_1_→S_0_ and $S_{d}^{L}∼10^{-40}$ esu^2^∙cm^2^ for T_1_→S_0_ transitions.

Equations 7, 8 describe the “spin strengths” of the exchange interaction between the donor and acceptor molecules. $\langle \psi^{*}J^{*}\left\|S\right\|\psi J\rangle$ represents the reduced matrix elements of the spin operator from the Ln^3+^ side, while $s_{m}$ is the spin operator coupled with the z-component of the dipole operator $\mu_{z}$ from the ligand side (e Silva and Malta, 1997; Malta and Gonçalves e Silva, 1998; Carneiro Neto et al., 2019).

In these equations there are several important quantities to consider. These include $\Omega_{\lambda }^{FED}$ , which are intensity parameters that take into account the forced electric dipole (FED) mechanism as described in the Judd-Ofelt theory (Judd, 1962; Ofelt, 1962), and the squared reduced matrix elements ${\langle \psi^{*}J^{*}\left\|U^{\left(\lambda \right)}\right\|\psi J\rangle }^{2}$ , whose values can be found in reference (Carnall et al., 1978). $\langle f\left\|C^{\left(\lambda \right)}\right\|f\rangle$ are equal to −1.366, 1.128, and −1.270 for λ = 2, 4, and 6, respectively. The $\langle r^{\lambda }\rangle$ and $\left(1-\sigma_{\lambda }\right)$ are 4f radial integrals and 4f shielding factors, respectively (Carnall et al., 1983; Edvardsson and Klintenberg, 1998; Smentek, 1998; Malta, 2008; Carneiro Neto and Moura, 2020).

Last but not least, the F parameter is the spectral overlap factor that considers the energy mismatch between the donor and acceptor transitions. To calculate it, we can use the fact that the bandwidth at half-height for the ligands ( $\gamma_{L}\approx 3000$ cm^−1^) is much larger than for Ln^3+^ ( $\gamma_{\mathrm{Ln}}\approx 300$ cm^−1^). Therefore, in the condition of $\gamma_{L}\gg \gamma_{\mathrm{Ln}}$ , we can use a simplified formula for the F parameter (Smentek and Kędziorski, 2010; Carneiro Neto et al., 2019):

$$F=\frac{G\left(\delta ,T\right)}{\hbar \gamma_{L}}\sqrt{\frac{\ln\left(2\right)}{\pi }}e^{-{\left(\frac{\Delta }{\hbar \gamma_{L}}\right)}^{2}\ln\left(2\right)}\;with\;G\left(\delta ,T\right)\left\{\begin{array}{c} 1\;if\;\delta \geq 0\\ e^{\left(\frac{\Delta }{k_{B}T}\right)}\;if\;\delta <0 \end{array}\right.$$ (9)

where the Δ is the band maximum energy difference between the donor state ( D ) and Ln^3+^ acceptor state, $\Delta =E_{D}-E_{\mathrm{Ln}}$ . The temperature dependence is given by the $G\left(\delta ,T\right)$ , for which a Boltzmann energy barrier $\exp\left(\Delta /k_{B}T\right)$ is activated when Δ<0 , where T is the temperature and $k_{B}$ is Boltzmann’s constant.

To predict the population of each level (Figure 2) after estimating the IET rates theoretically, a rate equations model can be employed (Grant, 1971). This model consists of a set of ordinary differential equations which take into account all the rates (including decay and IET rates) involving different levels. Each level is described using the general form:

$$\frac{dN_{i}\left(t\right)}{dt}=\underset{j=1}{\sum }W_{j\to i}N_{j}\left(t\right)-\underset{j=1}{\sum }W_{i\to j}N_{i}\left(t\right)$$ (10)

where $N_{i}\left(t\right)$ and $N_{j}\left(t\right)$ represent the population of levels $\left|i\right.\rangle$ and $\left|j\right.\rangle$ respectively, at any given time “t”. The energy transfer rate from level $\left|j\right.\rangle$ to $\left|i\right.\rangle$ is denoted by $W_{j\to i}$ , while the reverse process is represented by $W_{i\to j}$ . When dealing with a system that has “n” levels, the rate equations model will require “n” coupled ordinary differential equations to be solved. The rate equation modelling procedure commonly employs a normalized population, where the sum of all populations must be equal to 1.

There are two approaches for solving the system of equations: analytical and numerical. The analytical method assumes that the system is in a steady-state regime (Carneiro Neto et al., 2019; Kasprzycka et al., 2020), which means that the derivatives of the populations are equal to zero. On the other hand, the numerical approach solves and propagates the equations through time. In this case, it is important to ensure that the system reaches the steady-state regime by the end of the numerical simulation (Carneiro Neto et al., 2019; Carneiro Neto et al., 2021). By solving these equations, we can obtain a quantitative understanding of the population of each energy level and the kinetics of the energy transfer process.

## 4 Synthesis, structural features, and thermal stability of coordination compounds Cat[LnL_4_], [LnL_3_Q], and [Ln(HL^2^)_3_(NO_3_)_3_] based on phenyl-containing carbacylamidophosphates

Carbacylamidophosphates (CAPhs) have been known since the 1960s when A. Kirsanov with his research group presented the so-called phosphazo reaction (Kirsanov and Makitra, 1956). Since the 1990s, these compounds have been used as ligands for different metal ions. And nowadays, the Cambridge Structural Database contains over two thousand structures with the –C(O)N(H)P(O)= system.

**FIGURE 3:**
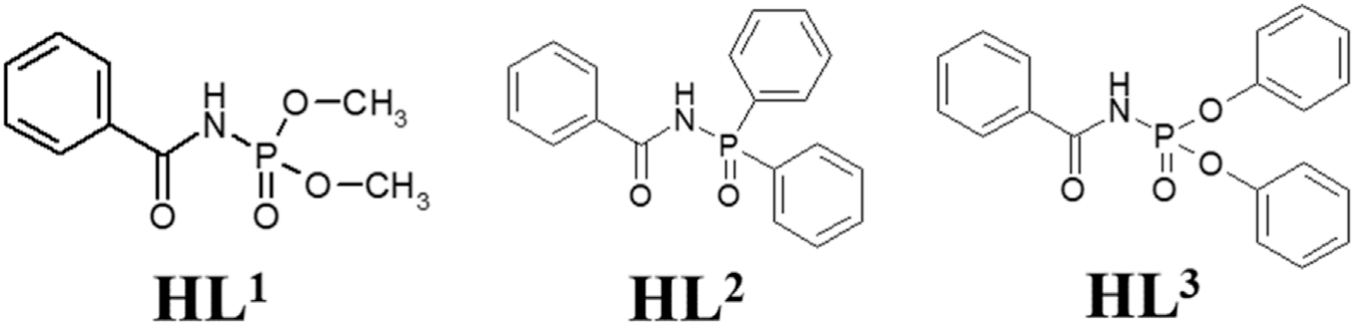
The structural formula of phenyl containing CAPhs.

**FIGURE 4:**
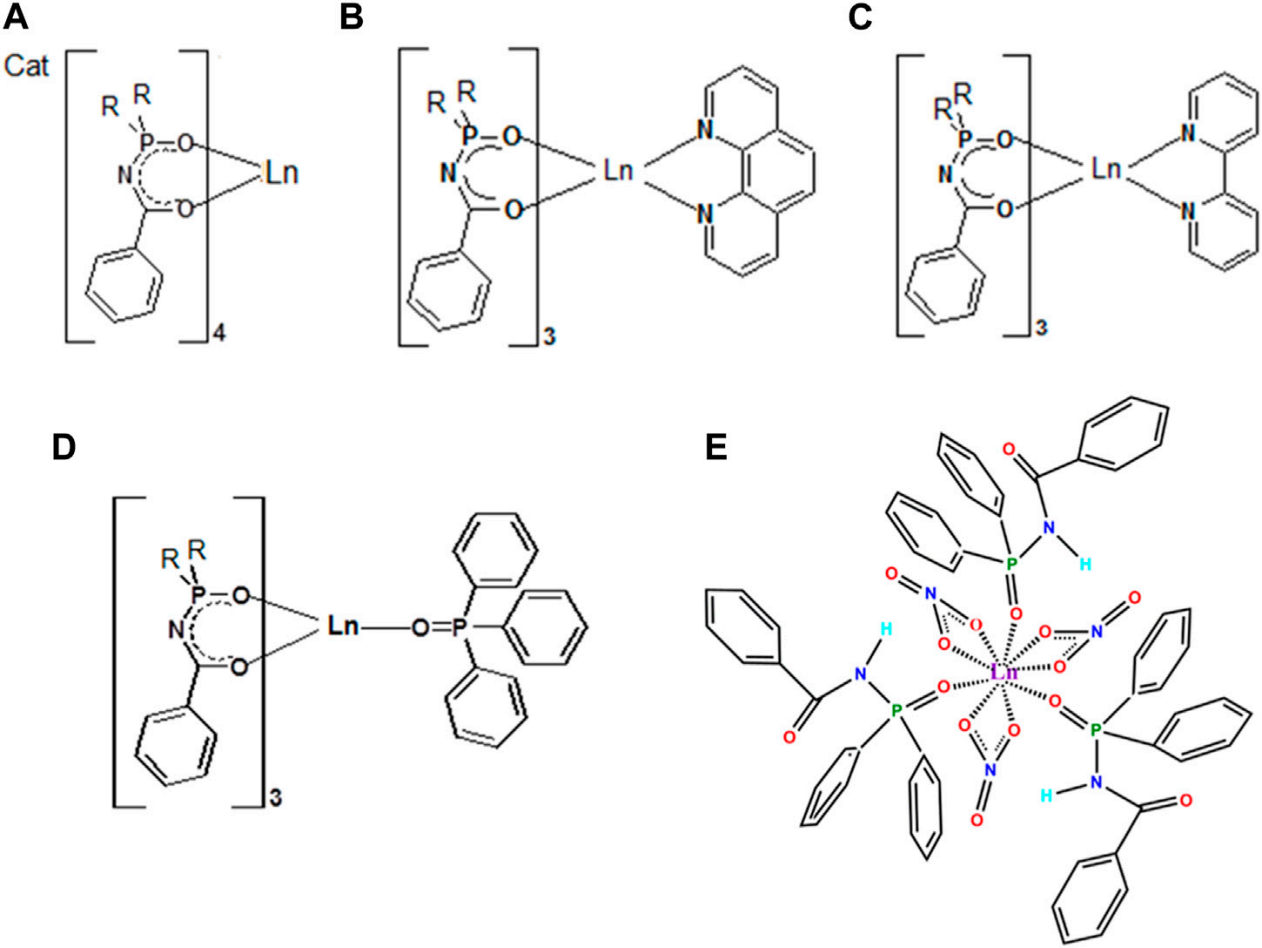
The structural formula of typical Ln^3+^ coordination compounds based on phenyl-containing CAPhs: Cat[LnL_4_] **(A)**, [LnL_3_Q] **(B-D)** and [Ln(HL^2^)_3_(NO_3_)_3_] **(E)**.

Herein we will limit ourselves to three representatives of CAPhs (Figure 3) with phenyl substitutes in different positions, which have been intensively studied recently as Eu^3+^ and Tb^3+^ luminescence sensitizers. The most typical CAPh based coordination compound types, Cat[LnL_4_], [LnL_3_Q], and [Ln(HL^2^)_3_(NO_3_)_3_] will be discussed (Figure 4).

Generally, the two types of Ln^3+^ coordination compounds may be obtained, based on β-diketones or CAPhs, depending on the ratio of metal ions to the ligands anions in the product: ternary compounds with a ratio Ln:Ligand = 1:3 or tetrakis-compounds with a ratio Ln:Ligand = 1:4. Additionally, some CAPhs, being in neutral form, can bind metals and create ternary coordination compounds of general formula [Ln(HL)_3_A_3_] (where A is a nitrate or chloride anion) (Amirkhanov et al., 2014).

### 4.1 Tetrakis-coordination compounds Cat[LnL_4_]

Coordination compounds of general formula Cat[LnL_4_], where L = β-diketonate or CAPh and where the cationic counterparts are typically alkali metal ions or positively charged N, P-organic cation, have been extensively studied recently (see (Kariaka et al., 2022) and references therein). In the structure of tetrakis-compounds, a lanthanide ion is surrounded by four deprotonated ligands forming an LnO8 environment. Such compounds are formed by the self-assembly principle—the cationic counterparts should coincide by size and/or create short contact with the ligand to form the coordination compound of the mentioned type. By using such cations as sodium, caesium, tetraethylammonium, and tetraphenyl phosphonium, the following coordination compounds have been obtained based on **HL** ^**1-3**^ ligands: Cs[LnL^1^]_4_, NEt_4_[LnL^1^]_4_, NEt_4_[LnL^3^]_4_, and PPh_4_[LnL^3^]_4_ (Kariaka et al., 2016a; Kariaka et al., 2017; Kariaka et al., 2018; Kariaka et al., 2022).

To obtain NEt_4_[LnL^1^]_4_, NEt_4_[LnL^3^]_4_, and PPh_4_[LnL^3^]_4_ the following synthetic route can be applied: a solution of LnCl_3_·nH_2_O (1 mmol) in 2-propanol, previously boiled with a dehydrating agent HC(OEt)_3_, is mixed with the solution of NaL (4 mmol) in acetone. Then the CatCl (1 mmol) solution in 5 mL of acetone is added. The resulting mixture is heated to boiling point and then cooled to room temperature. After filtering out the precipitated NaCl, the remaining liquid is left to slowly evaporate. In a day, well-faceted crystals of the target compounds appear. The crystals are filtered off, washed with cold 2-propanol, and dried in air. The crystals of NEt_4_[LnL^1^]_4_ coordination compounds can be obtained with a yield of around ∼70% for lanthanides with bigger radii (La–Dy) and ∼50% for Ho–Yb (Kariaka et al., 2022). These compounds are well soluble in DMSO, alcohols, acetone, acetonitrile, and dichloromethane, and under heating soluble in benzene, and insoluble in water. The melting points of the coordination compounds are in the range of 112–143°С. The NEt_4_[LnL^3^]_4_ and PPh_4_[LnL^3^]_4_ can be obtained with a yield of ∼80%. NEt_4_[LnL^3^]_4_ compounds are well soluble in DMSO, methanol, acetone, acetonitrile, dichloromethane, not very soluble in benzene and 2-propanol, and insoluble in water. The melting points of these compounds are in the range of 110–135°С. The PPh_4_[LnL^3^]_4_ compounds are soluble in DMSO, methanol, acetone, and CH_2_Cl_2_, and insoluble in nonpolar organic solvents and water. The melting points are in the range of 220–265°С.

To obtain coordination compounds Cs[LnL^1^]_4_ the following synthetic route can be applied: a solution of LnCl_3_·nH_2_O (1 mmol) in 2-propanol, previously boiled with a dehydrating agent HC(OEt)_3_, is mixed with a solution of NaL (3 mmol) in acetone. Then CsL (1 mmol) solution in 5 mL of acetone is added. The resulting mixture is heated to reflux and then cooled to room temperature. The NaCl that has precipitated is filtered out, and the resulting liquid is left in a vacuum desiccator over CaCl_2_ to dry out. The next day the obtained crystals of the compounds are filtered off, washed with cold 2-propanol, and dried in air. Yield: ∼70% for lanthanides with bigger radii (La–Dy) and ∼50% for Ho–Yb tetrakis-compounds. The coordination compounds are soluble in DMSO, alcohols, and acetone, and insoluble in non-polar organic solvents and H_2_O. The melting points of the compounds are in the range of 210–240°С.

Ligand **HL** ^**3**^ does not form crystals of tetrakis-coordination compounds with an alkali ion in its outer-sphere, and **HL** ^**1**^ does not form tetrakis-coordination compounds with the sodium cation, apparently due to steric complications during contact of oxygen atoms of phosphoryl groups with those metal cations. **HL** ^**2**^ does not form tetrakis-compounds at all, presumably due to the too-close location of bulky phenyls to the chelating fragment.

The thermal decomposition of the phenyl containing CAPhs based Eu^3+^ tetrakis-compounds starts over 200°С, and after heating them to 600°С results in a weight loss of 51%—73%. The obtained residue is usually a mixture of europium phosphates. The total weight loss is less than for lanthanide β-diketonates, for which the total weight loss in this temperature range is usually over 80% (Bruno et al., 2009; Stilinović and Kaitner, 2009; Yi et al., 2012; Adati et al., 2019; Borges et al., 2019), due to their high volatility or formation of lanthanide oxides as residues. Tetrakis-compounds Cs[EuL^1^]_4_ and PPh_4_[EuL^3^]_4_ melt at a temperature of 240°С and decompose immediately after melting. The coordination compounds with tetraethylammonium cation have almost the same temperatures of decomposition; however, they have significantly lower melting points. Thus NEt_4_[EuL^1^]_4_ and NEt_4_[EuL^3^]_4_ exist in a liquid state in the temperature ranges 139–200 and 119–250°С, respectively.

The typical polyhedra of Ln^3+^ ions in the CAPh-based tetrakis-compounds are distorted square antiprism (D_4d_) and triangular dodecahedron (D_2d_). These types of polyhedra are also typical for Ln^3+^ tetrakis-β-diketonates (Bruno et al., 2009; Lunstroot et al., 2009; Stilinović and Kaitner, 2009). In the tetrakis-structure, the outer-sphere cations can influence the Ln^3+^ ions polyhedral geometry, due to forming short contacts with the CAPh ligands. Specifically, they interact with the phosphoryl and ester oxygen atoms of the CAPh ligands.

### 4.2 Ternary mixed ligand coordination compounds [LnL_3_Q]

In β-diketones or CAPhs-based Ln^3+^ ternary coordination compounds, the lanthanide ion coordination sphere cannot be saturated by three of these ligands. As a result, the ternary compounds contain solvents coordinated with the lanthanides. To remove the solvents and to improve the luminescence properties of the coordination compounds, ancillary ligands, such as 2,2-bipyridine (bipy), 1,10-phenanthroline (phen), triphenylphosphine oxide (TPPO), etc., are used.

The [LnL_3_Q] coordination compounds with CAPhs can be obtained according to the following synthetic route: a solution of Ln (NO_3_)_3_·nH_2_O (1 mmol) in 2-propanol, previously boiled with a dehydrating agent HC(OEt)_3_, is mixed with the solution of NaL (3 mmol) in acetone. Then 1 mmol of solid bipy or phen or TPPO is added to the mixture. The resulting mixture is boiled for some minutes to complete the dissolution of the ancillary ligand and then cooled down to room temperature. After 15 min, the precipitated NaNO_3_ is filtered off. The resulting clear solution is left to stand at ambient temperature for slow evaporation of the solvents. In a day, the coordination compounds precipitate as powders or crystals. The target precipitates are filtered off, washed with cold 2-propanol, and finally dried in air.

In the [LnL_3_bipy] and [LnL_3_phen], a lanthanide ion is bonded by six oxygen atoms of CAPhs and two nitrogen atoms of the ancillary ligand, forming an LnO_6_N_2_ environment. The typical polyhedra of lanthanide ions in ternary mixed ligand coordination compounds LnL_3_bipy and LnL_3_phen with phenyl-containing CAPhs are distorted square antiprisms (D_4d_) and triangular dodecahedra (D_2d_). In the LnL^1^_3_TPPO, the central ion is surrounded by seven oxygen atoms, and the lanthanide ion polyhedron is interpreted as a distorted pentagonal bipyramid (D_5h_) (Kuzmina et al., 2020). As the CAPh ligands are bulkier compared with β-diketones, the mixed ligand compounds with CAPhs contain one TPPO ligand, while for β-diketones-based ones, both types [LnL_3_TPPO] and [LnL_3_ (TPPO)_2_] can be obtained (Nakajima et al., 2016; Zhao et al., 2018).

The mixed ligand coordination compounds are thermally stable up to 200°С and even beyond (Table 1). The LnL^1^_3_TPPO has the lowest decomposition temperature (200°С). The highest decomposition temperature (280°С) is a characteristic of [EuL^2^_3_bipy] and [EuL^3^_3_phen] coordination compounds. The total compounds’ weight loss in the temperature range of 20–800°С does not exceed 70%, being smaller compared to mixed ligand β-diketonates. None of the mixed ligand compounds decompose immediately after melting and exist in fusion. The compound EuL^3^_3_phen has the lowest melting point (82°С) and the biggest temperature interval of the coordination compound existence in the liquid state (almost 200°С). The melting points and temperature of decomposition do not vary significantly depending on the lanthanide ion. No tendency in melting points and temperature of decomposition exists depending on ancillary ligand type in the [LnL_3_bipy] and [LnL_3_phen] coordination compounds. Thus, [LnL^1^_3_bipy] and [LnL^1^_3_phen] have similar melting and decomposition temperatures, the compounds [LnL^2^_3_bipy] and [LnL^2^_3_phen] differ in their melting points, while [EuL^3^_3_bipy] and [EuL^3^_3_phen] differ significantly in both their melting points and temperatures of decomposition.

**TABLE 1:** Melting points, the temperatures of decomposition, and weight losses of ternary coordination compounds.

Coordination compound	Melting point, °С	Temperature of decomposition, °С	Weight loss in a temperature range of 20–800°С, %
[EuL^1^_3_phen]	200	250	46
[TbL^1^_3_phen]	200	240	56
[EuL^1^_3_bipy]	190	230	54
[TbL^1^_3_bipy]	180	230	54
[NdL^1^_3_TPPO]	158	200	67*
[TbL^1^_3_TPPO]	164	200	67*
[LuL^1^_3_TPPO]	160	200	61*
[EuL^2^_3_phen]	190	260	70
[TbL^2^_3_phen]	200	250	68
[EuL^2^_3_bipy]	130	280	64
[TbL^2^_3_bipy]	135	260	63
[EuL^3^_3_phen]	82	280	66
[EuL^3^_3_bipy]	200	210	71

* In a temperature range of 20–600°С

### 4.3 Coordination compounds [Ln(HL^2^)_3_(NO_3_)_3_]

Compounds [Ln(HL^2^)_3_(NO_3_)_3_] can be obtained as follows: a solution of Ln(NO_3_)_3_·nH_2_O (1 mmol) in acetone is mixed with the solution of HL^2^ (3 mmol) in acetone. The solvent is completely evaporated from the resulting mixture. Then hexane is added to the obtained oily residue, and this mixture is mixed for 2 hours to obtain a solid precipitate. The obtained precipitate of the target compounds is filtered off, washed with hexane, and dried in air. The coordination compounds are well soluble in acetone, CH_2_Cl_2_, and methanol, slightly soluble in 2-propanol and benzene, and insoluble in hexane and water. The melting points of the compounds are in the range of 122–140°С. By spectral studies and elemental analysis, it was established that the CAPh ligands are coordinated to lanthanides in a monodentate manner via the oxygen atom of the phosphoryl group, while the nitrate anions are coordinated in a bidentate manner (Legendziewicz et al., 2000). Thus, the lanthanides have an LnO_9_ coordination environment. Thermal gravimetric studies have shown that the compound [Eu(HL^2^)_3_(NO_3_)_3_] starts to decompose at 140°С and the total weight loss in the temperature range 20–800°С equals 71%.

## 5 Absorption and phosphorescence spectra of phenyl-containing CAPh based Ln^3+^ coordination compounds

**FIGURE 5:**
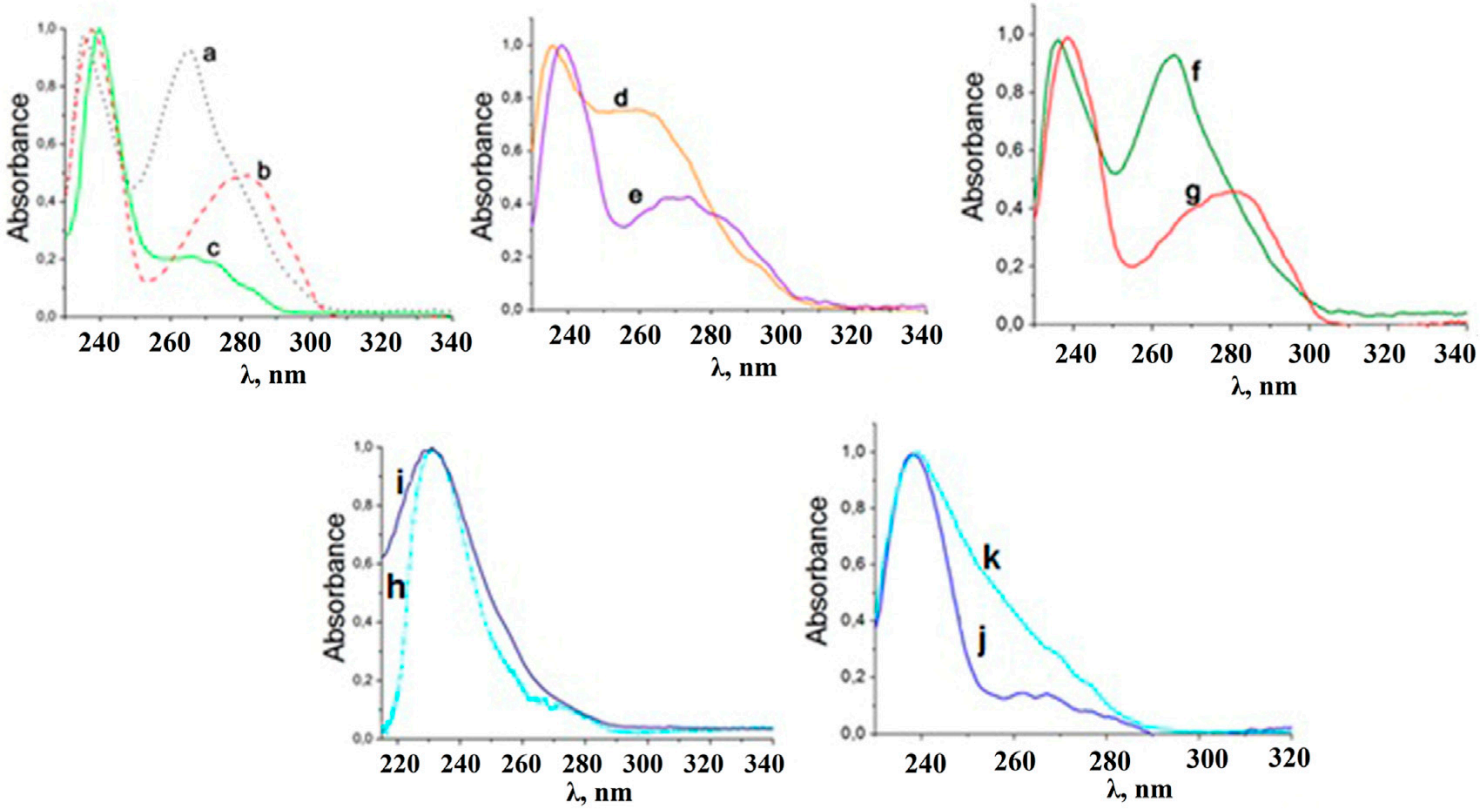
Normalized absorption spectra of [LnL^1^_3_Phen] (a), [LnL^1^_3_bipy] (b), [LnL^1^_3_TPPO] (c), [LnL^2^_3_Phen] (d), [LnL^2^_3_bipy] (e), [LnL^3^_3_Phen] (f), [LnL^3^_3_bipy] (g), solutions in CH_2_Cl_2_, Cs[LnL^1^_4_] (h), and NEt_4_[LnL^1^_4_] (i), solutions in CH_3_CN and NEt_4_[LnL^3^_4_] (j), PPh_4_[LnL^3^_4_] (k) solutions in CH_2_Cl_2_ at 298 K.

In the UV region, the absorption spectra of the synthesized mixed ligand coordination compounds (Figure 5) are characterized by the presence of the two absorption bands with maxima at 235 and 265 nm for the compounds with phen, 238 and 275–280 nm for the compounds with bipy, and 240 and 265 nm for [LnL^1^_3_TPPO].

The absorption spectra of the obtained tetrakis-coordination compounds contain one intense band with a maximum of 236–238 nm and a shoulder at 280 nm (Figure 5). The absorption bands in the spectra appear due to π→π* transitions of the ligands.

Considering the lowest energy edge of the absorption spectra of the coordination compounds, it can be assumed that the CAPhs lowest excited singlet state is located near 35 500 cm^−1^, the lowest excited singlet state of bipy and phen is near 33 100 cm^−1^, and for the TPPO ligand it is near 34 500 cm^−1^.

The lowest ligands triplet states positions Е(T_1_) in the CAPh-based compounds, estimated from the phosphorescence spectra of La^3+^ or Gd^3+^ compounds using the zero-phonon line, as well as for lanthanide coordination compounds based on phenyl containing β-diketonates, are given in Table 2. It is noteworthy that phenyl-containing CAPhs have higher Е(T_1_) compared with phenyl-containing β-diketones by nearly 6,000 cm^−1^. It is also worth noting that in mixed ligand coordination compounds, the Е(T_1_) is variable depending on the combination of the ligands. For example Е(T_1_) of phen in CAPh-based compounds [LnL_3_phen] varies from 21,552 to 22,371 cm^−1^ depending on the CAPh ligand. In compounds [Ln(bzac)_3_·2H_2_O] and [Ln(bzac)_3_phen], the Е(T_1_) of bzac ligand differs depending on the ancillary ligand, being equal to 21,550 and 20,202 cm^−1^, respectively. Comparing Е(T_1_) in the tetrakis-compounds, it can be concluded that the lowest ligands’ triplet states position also depends slightly on the nature of the cation.

**TABLE 2:** The lowest ligands triplet state energies Е(T_1_) and the energy gaps ΔE (in cm^−1^) between T_1_ and ^5^D_J_ levels.

Coordination compound	Е(T_1_)	ΔE (T_1_-^5^D_0_)	ΔE (T_1_-^5^D_1_)	ΔЕ (Т_1_-^5^D_4_)
Cs[LnL^1^_4_]	27 100	9,800	8,082	6,600
NEt_4_[LnL^1^_4_]	26 955	9,654	7,937	6,455
[LnL^1^_3_phen]	22 371	5,071	3,353	1871
[LnL^1^_3_bipy]	22 883	5,583	3,865	2,383
[LnL^1^_3_TPPO]	24 390	7,090	5,372	3,890
[Ln (HL^2^)_3_(NO_3_)_3_]	26 178	8,878	7,160	5,678
[LnL^2^_3_phen]	21 552	4,252	2,534	1,052
[LnL^2^_3_bipy]	22 883	5,583	3,865	2,383
NEt_4_[LnL^3^_4_]	26 385	9,085	7,367	5,885
PPh_4_[LnL^3^_4_]	26 954	9,654	7,936	6,454
[LnL^3^_3_phen]	21 739	4,439	2,721	1,239
[LnL^3^_3_bipy]	22 831	5,531	3,813	2,331
[Ln(bzac)3·2H2O] Junior et al. (1997)	21 550	4,250	2,532	1,050
[Ln(bzac)_3_phen] Junior et al. (1997)	20 202	2,902	1,184	−298
[Ln(dbm)_3_·2H_2_O] Teotonio et al. (2012)	20 534	3,234	1,516	34

Hbzac—benzoylacetone; Hdbm—dibenzoylmethane.

## 6 General interpretations of the Eu^3+^ and Tb^3+^ β-diketonates photoluminescence

β-diketones were among the first ligands discovered to be suitable sensitizers for the Eu^3+^ ion (Yuster and Weissman, 1949). These ligands form stable coordination compounds with lanthanide ions, allowing for efficient sensitization due to the small distance between the sensitizer and the ion. Furthermore, energy transfer from ligands to the metal in lanthanide β-diketonates occurs mainly through the triplet state, and the energy transfer from the excited singlet level of the ligand is often less relevant. As a result, many Eu^3+^ β-diketonate compounds exhibit intense luminescence. However, these ligands are often not the best sensitizers for Tb^3+^ ions since their triplet state has rather low energy, leading to an effective backward energy transfer from the Tb^3+^ (Yang et al., 1994a).

In search for effective luminescent lanthanide coordination compounds based on β-diketones, the main approach is to modify the β-diketone substituents. The luminescence properties of lanthanides can also be tuned by selecting suitable ancillary ligands in mixed ligand compounds or changing the outer-sphere cations in tetrakis-compounds. The luminescence of Eu^3+^ β-diketonate coordination compounds follows certain regularities based on their structure. Typically, tris- (or ternary) coordination compounds exhibit lower emission intensity, while the intensities of tetrakis-compounds are usually higher. Addition of an ancillary ligand to the coordination sphere of ternary coordination compounds generally increases their emission intensity. However, due to a significant energy gap between the resonance level of Eu^3+^ and the triplet of the ligands, the energy transfer is inefficient in coordination compounds with aliphatic β-diketones, such as acetylacetone (Hacac), trifluoroacetylacetone (Htfac), or hexafluoroacetylacetone (Hhfac), resulting in poor emission. This was discussed in the work by Filipescu and collaborators (Filipescu et al., 1964). Using a combination of both aromatic and aliphatic substituents in the β-diketonate ligands, such as benzoylacetone (Hbzac), benzoyltrifluoroacetone (Hbtfac), and thenoyltrifluoroacetone (Htta), can lead to compounds with more efficient emission than those containing symmetrical ligands like dibenzoylmethane (Hdbm), dithionylmethane (Hdtm), Hacac, or Hhfac. The increase in emission intensity in such systems is attributed to an increase in anisotropy around the Eu^3+^ ion (Filipescu et al., 1964; Lima et al., 2013).

The quantum yield of coordination compounds with fluorinated ligands is higher than non-fluorinated analogues. Among europium β-diketonates, a well-known coordination compound with good luminescent characteristics is [Eu (tta)_3_Phen]. S. B. Meshkova and co-authors have studied the photoemission of β-diketonates with perfluoroalkyl chains (Topilova et al., 1997; Meshkova et al., 2000). They have shown that the intensity and quantum yield of lanthanide luminescence, as well as the absorption capacity of the ligands, increase with the increasing number of carbon atoms in the chain. Such dependence was observed when the chain was increased to the size of C_6_F_13_ and was explained by the formation of a reliable hydrophobic shell around the metal ions.

Among Tb^3+^ coordination compounds, tris-compounds with 4,4′-difluorobenzoylmethane (Hfbm), di-4,4′-dimethoxybenzoylmethane (Hdmbm) (Filipescu et al., 1964), and acetylacetone (Yang et al., 1994a) demonstrated intensive emission. In order to obtain luminescent Tb^3+^ β-diketonates with aromatic substituents, compounds with 1-indoleacetylacetone and 3-indoleacetylacetone (Wu et al., 1992a; Wu et al., 1992b) were synthesized, since the energy of the triplet level of indole is higher than that of the phenyl group.

In the works (Yang et al., 1994a; Yang et al., 1994b), it was also noted that the rigid planar structure of chelating metallocycles contributes to the growth of metal-center emission sensitized by ligands since such a structure improves the energy transfer from the ligand to the metal.

Eu^3+^ and Tb^3+^ β-diketonate coordination compounds are usually excited by UV light. However, there is a need to find coordination compounds that can be excited by visible light for their use as luminescent labels. In this sense, several studies have focused on this search (Werts et al., 1999; Jiang et al., 2010; Ma and Wang, 2010; Reddy et al., 2013; Sun et al., 2015). Additionally, some Ln^3+^ β-diketonate have been reported to have low photostability when exposed to UV irradiation (Pagnot et al., 2000; Gameiro et al., 2001; Nockemann et al., 2005; Lima et al., 2006; Lima et al., 2009; Songzhu et al., 2010; Kai et al., 2011).

Another important indicator of the emission, which determines the practical interest in it, is the luminescence decay time (τ_exp_). Unlike the quantum yield, which reflects emission quenching in the entire ligand-metal system, the luminescence lifetime characterizes non-radiative processes occurring only on the metal ion. Fluoride substituents in β-diketones have a positive effect on the value of the emission lifetime, increasing it almost twice compared to non-fluorinated analogues. The luminescence lifetime of Eu^3+^ ternary-compounds is shorter than that of tetrakis-compounds at 77 K, but the inverse relationship is observed at room temperature.

The luminescence characteristics of some Eu^3+^ and Tb^3+^ β-diketonate are presented in Table 3. As can be seen from the table, most β-diketonates of Eu^3+^ and Tb^3+^ show emission with a lifetime of up to a millisecond, and various quantum yields: from fractions of a percent to almost one hundred percent values (70%–85%). But as noted, the value of the quantum yield gives only a partial idea of the luminescence intensity of compounds, because the latter depends not only on the quantum yield but also on the amount of energy absorbed by the coordination compound (Binnemans et al., 2005).

**TABLE 3:** Luminescence decay lifetime, intrinsic and overall quantum yields of some Eu^3+^ and Tb^3+^ β-diketonates.

Coordination compound	τ _exp,_ μs	$Q_{\mathrm{Ln}}^{\mathrm{Ln}}$ , %	$Q_{\mathrm{Ln}}^{L}$ , %	References
[Eu(bzac)_3_(H_2_O)_2_]	300	21 (5)	—	Junior et al. (1997), de Sá et al. (2000)
[Eu(bzac)_3_phen]	410	28 (8)	—	Junior et al. (1997), de Sá et al. (2000)
[Eu(bzac)_3_phenNO]	855	—	27	Junior et al. (1997), de Sá et al. (2000)
[Eu(btfa)_3_(H_2_O)_2_]	380	24	—	de Mello Donegá et al. (1997)
[Eu(btfa)_3_phenNO]	670	56	—	de Mello Donegá et al. (1997)
[Eu(tta)_3_(H_2_O)_2_]	260	29	23	Malta et al. (1998)
[Eu(tta)_3_(DBSO)_2_]	714	70	85	e Silva et al. (2000)
[Eu(tta)_3_phen]	976	63	69	e Silva et al. (2000)
[Tb(tta)_2_(NO_3_)(TPPO)_2_]	—	—	38	Teotonio et al. (2010)
[Eu(dbm)_3_H_2_O]	230–350	25	—	Teotonio et al. (2012)
[Eu(dbm)_3_DMA]	—	59	45	Teotonio et al. (2012)
[Eu(dbm)_3_DMF]	—	60	43	Teotonio et al. (2012)
[Eu_2_(btb)_3_(C_2_H_5_OH)_2_(H_2_O)_2_]	366	30	—	Shi et al. (2013)
[Eu_2_(btb)_3_(phen)_2_]	906	78	—	Shi et al. (2013)
[Eu(nta)_3_(dmso)_2_]	—	—	75	Carlos et al. (2003)
[Eu(nta)_3_p-tolyl-DAB]	—	—	1.6	Carlos et al. (2005)
[C_4_mim][Eu(nta)_4_]	559	—	72–77	Bruno et al. (2009)
[C_4_mpyr][Eu(nta)_4_]	693	—	—	Bruno et al. (2009)
[NBu_4_][Eu(nta)_4_]	269	46	46	Bruno et al. (2009)
[Tb(ppi)_3_(H_2_O)_2_]	785	97	59	Biju et al. (2009)
[Tb(ibpi)_3_(C_2_H_5_OH)(H_2_O)]	920	96	72	Biju et al. (2009)
[Eu(ppi)_3_(H_2_O)_2_]	259	15	0.3	Biju et al. (2009)
[Eu(ibpi)_3_(C_2_H_5_OH)(H_2_O)]	261	16	0.5	Biju et al. (2009)
[Eu(ETFMCTFBD)_3_phen]	—	34	—	He et al. (2010)
[Eu(tmh)_3_TPPO]	760	80	—	Yanagisawa et al. (2015)
[Eu(bmdm)_3_TPPO]	450	55	—	Monteiro et al. (2011)
[Tb(dbm)_3_TPPO]	21	—	—	Silva et al. (2013)
[Tb(tmh)_3_TPPO]	840	—	—	He et al. (2010)
[Eu(hfa)_3_(TPPO)_2_]	800	65	51	Nakajima et al. (2016)
[Eu(hfaс)_3_(H_2_O)_2_]	220	19	2.6	Eliseeva et al. (2008)
[Eu_2_(hfaс)_6_(O(CH_2_)_2_NHMe_2_)_2_]	990	71	58	Eliseeva et al. (2008)
[Tb(thd)_2_(O(CH_2_)_2_NMe_2_)]	680	—	32	Eliseeva et al. (2008)
[Tb(hfaс)_3_(H_2_O)_2_]	540	—	27	Eliseeva et al. (2008)
[Tb_2_(hfaс)_6_(O(CH_2_)_2_NHMe_2_)_2_]	too low	—	0.04	Eliseeva et al. (2008)
[Tb(thd)_3_]	460	—	40	Eliseeva et al. (2008)
[Eu(tfi)_3_(H_2_O)_2_]	45	12	—	Li et al. (2012)
[Eu(tfi)_3_bipy]	84	18	—	Li et al. (2012)
[Eu(tfi)_3_phen]	128	25	—	Li et al. (2012)
[Eu(bfpd)_3_(H_2_O)_2_]	399	37	2	Divya et al. (2011)
[Eu(nfpd)_3_(H_2_O)_2_]	400	42	7	Divya et al. (2011)
[Eu(bpfpd)_3_(H_2_O)_2_]	376	32	7	Divya et al. (2011)
[Eu(bfpd)_3_TBNPO]	769	70	19	Divya et al. (2011)
[Eu(nfpd)_3_TBNPO]	790	73	28	Divya et al. (2011)
[Eu(bpfpd)_3_TBNPO]	877	71	43	Divya et al. (2011)
[Eu(pbi)_3_(H_2_O)(C_2_H_5_OH)]	250	26	—	Biju et al. (2006)
[Eu(pbi)_3_bipy]	978	68	—	Biju et al. (2006)
[Eu(pbi)_3_phen]	1,025	57	—	Biju et al. (2006)
[Eu(N-C1)_3_phen]	512	—	25	He et al. (2009)
[Eu(N-C2)_3_phen]	766	—	19	He et al. (2009)
[Eu(N-C3)_3_phen]	1,010	—	14	He et al. (2009)
[Eu(N-C5)_3_phen]	750	—	12	He et al. (2009)

PhenNO, 1,10-phenanthroline-N-oxide; Hbtfa, 1-phenyl-1,3-butanedione; Htta, thenoyltrifluoroacetone; DBSO, dibenzyl sulfoxide; DMA, dimethylacetamide; DMF, dimethylformamide; H2btb, 1,3-bis(4,4,4-trifluoro-1,3-dioxobutyl)phenyl; Hnta, 1-(2-naphtoil)-4,4,4- tri-fluroacetone; dmso, dimethyl sulfoxide; p-tolyl-DAB, 1,4-bis-p-tolyl-1,4-diaza-1,3-butadien; C4mim, 1-butyl-3-methylimidazole; C4mpyr, 1-butyl-l-3-methylpyridine; Hppi, 3-phenyl-4-propionyl-5-isoxazolone; Hibpi, 4-isobutyryl-3-phenyl-5-isoxazolone; HETFMCTFBD, 1-(9-ethyl-7-(trifluoromethyl)-9H-carbazol-2-yl)-4,4,4-trifluorobutane-1,3-dione; Htmh, 2,2,6,6-Tetramehylheptane-3,5-dione; Hbmdm, butyl methoxy-dibenzoyl-methane; Hhfa = Hhfaс, hexafluoroacetylacetonate; Hthd, dipivaloylmethane; Htfi, 2-(2,2,2-Trifluoroethyl)-1-indone; Hbfpd, 1-(1-phenyl)-3-(2-fluoryl) propanedione; Hnfpd, 1-(2-naphthyl)-3-(2-fluoryl)propanedione; Hbpfpd, 1-(4-biphenyl)-3-(2-fluoryl) propanedione; Hpbi, 3-phenyl-4-benzoyl-5-isoxazolone; TBNPO, 2,2′-bis(di-p-tolylphosphino)-1,1′-binaphtyloxide; Н(N-C1), 4,4,4-trifluoro-1-(9-methyl-9H-carbazol-3-yl)butane-1,3-dione; Н(N-C2), 4,4,4-trifluoro-1-(9-ethyl-9H-carbazol-3-yl)butane-1,3-dione; (N-C3), 4,4,4-trifluoro-1-(9-propyl-9H-carbazol-3-yl)butane-1,3-dione; (N-C5), 4,4,4-trifluoro-1-(9-pentyl-9H-carbazol-3-yl)butane-1,3-dione.

Not only the emission efficiency of β-diketonates of lanthanides is of interest. In view of applications of the coordination compounds in OLEDs, their luminescent color is an important factor to be considered, which depends on the emission band ratio intensity. In the emission spectra of Eu^3+^ β-diketonates, the band of the hypersensitive ^5^D_0_→ ^7^F_2_ transition dominates. The ratio of intensities of hypersensitive and magnetic dipole transitions I (^5^D_0_→^7^F_2_)/I (^5^D_0_→^7^F_1_) is in the range of 8–27 (Klink et al., 2000; Binnemans et al., 2005).

From a theoretical perspective, lanthanide-base β-diketonates have been widely studied in the literature (Moura et al., 2016; Carlos et al., 2005; Divya et al., 2011; Petrov et al., 2015; Arrué et al., 2021; Malta et al., 1997; Singh et al., 2017; Santos et al., 2020; dos Santos et al., 2010; Batista et al., 1998; Lima et al., 2020; Fu et al., 2019; Nigoghossian et al., 2022). Some computational studies have helped in understanding the molecular and electronic structures of lanthanide β-diketonates (Fu et al., 2019; Lima et al., 2020; Arrué et al., 2021) as well as in exploring their stability (Petrov et al., 2015). In addition, the influence of the covalency of Ln–O bonds in the so-called Ω_λ_ intensity parameters (sometimes referred to as Judd-Ofelt parameters) was also addressed using density functional theory (DFT) calculations (Moura et al., 2016). These kinds of studies have provided valuable insights into the design and development of new luminescent materials.

## 7 Photoluminescence of Eu^3+^ and Tb^3+^ complexes based on phenyl-containing carbacylamidophospates

In recent decades, coordination compounds based on hetero-substituted analogues of β-diketones, such as carbacylamidophosphates (CAPh) and sulfonylamidophosphates (SAPh), have been actively studied (Gawryszewska and Smolenski, 2014). From the point of view of obtaining luminescent materials, ligands of these types have some advantages over β-diketones. In particular, C=O vibrations (∼1,600 cm^−1^), which are present in β-diketones, are partially or completely replaced by less energetic P=O vibrations (∼1,250 cm^−1^) and S=O (∼1,350 cm^−1^); replacing the carbon atom with nitrogen in the chelating node of the ligands eliminates high-energy C–H oscillations; and the phosphoryl group, which is present in CAPhs and SAPhs, makes it possible to add one more “substitute-antenna”. Unlike many lanthanide β-diketonates, the amidophosphates form thermodynamically stable compounds which are not destroyed by UV, or even synchrotron irradiation (Gawryszewska and Smolenski, 2014).

The luminescence studies of coordination compounds based on CAPh ligands are currently limited. So far, only a few studies have investigated the luminescence properties of Eu^3+^ and Tb^3+^ coordination compounds with CAPh ligands. These studies include investigations on the luminescence of compounds with general compositions of Cat[LnL_4_]·xH_2_O (Amirkhanov et al., 1996; Sokolnicki et al., 1999; Tang et al., 2014; Kariaka et al., 2016a; Kariaka et al., 2017; Kariaka et al., 2018; Kariaka et al., 2022), [LnCl_3_(HL)_3_] and [Ln(NO_3_)_3_(HL)_3_] (Legendziewicz et al., 2000; Legendziewicz et al., 2001; Znovyak et al., 2009; Kariaka et al., 2016b), [LnL_3_Q] (Q = H_2_O, 2-propanol, 2,2-bipyridine, 1,10-phenanthroline, TPPO, 2-(1,3,4-oxadiazole-2-yl) pyridine) (Legendziewicz et al., 2001; Borzechowska et al., 2002; Oczko et al., 2003; Puchalska et al., 2008; Znovyak et al., 2009; Kariaka et al., 2014; Tang et al., 2014; Litsis et al., 2015; Kariaka et al., 2016b; Kariaka et al., 2016c; Nakajima et al., 2016; Yakovlev et al., 2018; Kariaka et al., 2020), [Na_2_LnL_4_(OTf)(DMF)] (Pham et al., 2020), Ln_5_L_10_(OH)_5_ (Kariaka et al., 2016d), binuclear Ln_2_L_3_phen_2_ (Horniichuk et al., 2021), and [Eu_2_(L)_2_(μ-Ph_2_POO)_2_(κ-OP(O)Ph_2_)_2_(CH_3_OH)_2_] (Pham et al., 2017). Some of these studies have also measured the luminescence decay lifetimes and emission quantum yields (overall and intrinsic), as shown in Table 4.

**TABLE 4:** Experimental luminescence characteristics of some phenyl-containing CAPh-based Eu^3+^ coordination compounds in crystalline state at room temperature.

Coordination compound	τ_exp,_ μs	$Q_{\mathrm{Ln}}^{\mathrm{Ln}}$ , %	$Q_{\mathrm{Ln}}^{L}$ , %	η_sens_, %	References
[C_2_mim][Eu(DETCAP)_4_]	2,700	76	30	39	Tang et al. (2014)
[C_4_mim][Eu(DETCAP)_4_]	2,700	75	49	65	Tang et al. (2014)
[Eu(Wo)_3_Q]	1,650	85	—	—	Yakovlev et al. (2018)
[Eu(Pip)_3_Phen]	1,580	89	—	—	Litsis et al. (2015)
[Eu_5_L^1^_10_(OH)_5_]	670	31	15	48	Kariaka et al. (2016d)
Cs[EuL^1^_4_]	3,200	92	37	40	Kariaka et al. (2016a)
NEt_4_[EuL^1^_4_]	2,200	62	26	42	Kariaka et al. (2017)
[EuL^1^_3_phen]	1810	73	40	55	Kariaka et al. (2016c)
[EuL^1^_3_bipy]	1910	79	42	53	Kariaka et al. (2016c)
[EuL^1^_3_TPPO]	1,300	80	—	—	Kuzmina et al. (2020)
[Eu(HL^2^)_3_(NO_3_)_3_]	1,660	78	44	56	Kariaka et al. (2016b)
[EuL^2^_3_phen]	1,540	76	32	42	Kariaka et al. (2014)
[EuL^2^_3_bipy]	1,410	67	34	51	Kariaka et al. (2014)
NEt_4_[EuL^3^_4_]	1800	66	37	56	Kariaka et al. (2017)
PPh_4_[EuL^3^_4_]	1,420	82	56	68	Kariaka et al. (2018)
[EuL^3^_3_phen]	1860	70	42	60	Kariaka et al. (2020)
[EuL^3^_3_bipy]	1970	78	46	59	Kariaka et al. (2020)
[Na_2_Eu(Lig)_4_(OTf)(DMF)]	1700	99	98	∼100	Pham et al. (2020)
[Eu_2_(LF)_3_phen_2_]	1880	91	—	—	Horniichuk et al. (2021)
[Eu_2_(Lig)_2_(Ph_2_POO)_4_(CH_3_OH)_2_]	950	39	—	—	Pham et al. (2017)

C_2_mim, 1-ethyl-3-methylimidazolium; C_4_mim, 1-butyl-3-methylimidazolium; DETCAP, diethyl-2,2,2-trichloroacetylphosphoramidate; HWo, dimethyl-N-trichloracetylamidophosphate; HPip, 2,2-trichloro-N-(dipiperidin-1-yl-phosphoryl)acetamide; HLig, N- (diphenylphosphoryl)pyrazine-2-carboxamide; OTf, trifluoromethanesulfonate; H_2_LF, tetramethyl N,N′-(2,2,3,3,4,4-hexafluoro-1,5-dioxopentane-1,5-diyl)bis(phosphoramidate).

**FIGURE 6:**
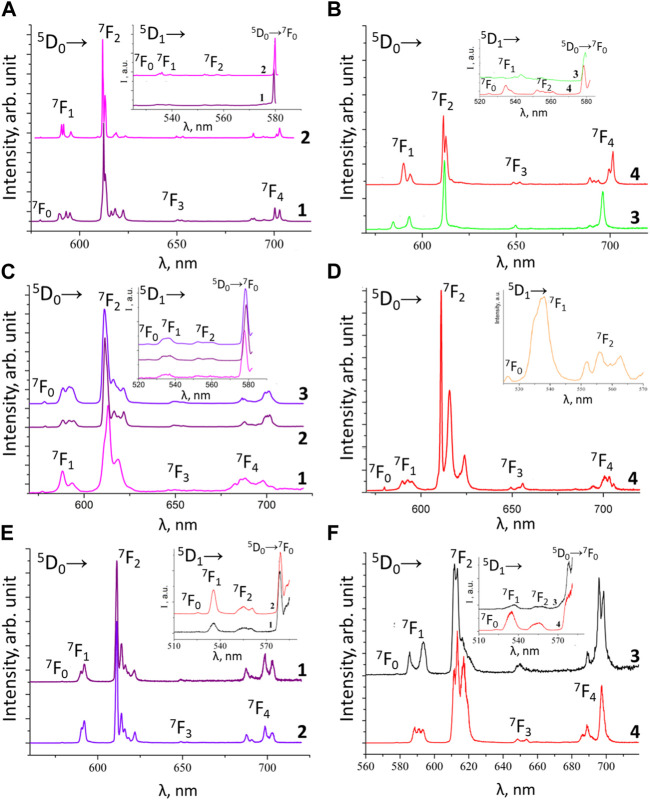
Low temperature (77 K) emission spectra of the europium compounds: **(A)** [EuL^1^_3_Phen] (1) and [EuL^1^_3_bipy] (2); **(B)** NEt_4_[EuL^1^_4_] (3) and Cs[EuL^1^_4_] (4); **(C)** [Eu(HL^2^)_3_(NO_3_)_3_] (1), [EuL^2^_3_Phen] (2) and [EuL^2^_3_bipy] (3); **(D)** [EuL^1^_3_TPPO] (4); **(E)** [EuL^3^_3_Phen] (1) and [EuL^3^_3_bipy] (2); **(F)** NEt_4_[EuL^3^_4_] (3) and PPh_4_[EuL^3^_4_] (4).

Herein we will limit ourselves to a more detailed discussion of the luminescence properties of phenyl-containing CAPh-based Eu^3+^ and Tb^3+^ coordination compounds (Figure 6), obtained in the experiment and using theoretical calculations. Among these coordination compounds, the highest value of $Q_{\mathrm{Ln}}^{\mathrm{Ln}}$ was observed for the Cs[EuL^1^_4_]. The overall emission quantum yield ( $Q_{\mathrm{Ln}}^{L}$ ) is the highest for PPh_4_[EuL^3^_4_]. In whole, considering the quantum yield measurement error (15%), quite close values of $Q_{\mathrm{Ln}}^{L}$ can be stated for all the phenyl containing CAPh-based Eu^3+^ compounds. Sensitization efficiency is in the range of 40%–68%. If compared with europium β-diketonates, the luminescence of CAPh-based Eu^3+^ compounds is characterized by noticeably longer luminescence lifetime and relatively high values of intrinsic quantum yields. The $Q_{\mathrm{Ln}}^{L}$ and η_sens_ values are comparable with those observed for europium β-diketonates.

The low temperature (77 K) luminescence spectra of the Eu^3+^ coordination compounds [EuL_3_bipy], [EuL_3_phen] (L = L^1^, L^2^, L^3^), Cat[EuL_4_] (L = L^1^, L^3^), [EuL^1^_3_TPPO], and [Eu(HL^2^)_3_(NO_3_)_3_] are shown in Figure 6. The insets in these figures show ^5^D_1_ emission and the transition ^5^D_0_→^7^F_0_ at 77 K.

The ^5^D_0_ luminescence spectra of these coordination compounds are characterized by the presence of narrow bands, corresponding to transitions ^5^D_0_→^7^F_J_ (J = 0–4) and the absence of the ligand’s fluorescence band. For all these Eu^3+^ coordination compounds, except for NEt_4_[EuL^3^_4_] and PPh_4_[EuL^3^_4_], the ^5^D_0_→^7^F_0_ transition band at 77 K is a symmetrical singlet. For the compounds NEt_4_[EuL^3^_4_] and PPh_4_[EuL^3^_4_], this band is of low intensity, which makes reliable analysis of it impossible. The intensities distributions and the ratios between the hypersensitive and the magnetic dipole transitions ^5^D_0_→^7^F_2_/^5^D_0_→^7^F_1_ (red/orange ratio) are given in Table 5.

**TABLE 5:** Photoluminescence intensity distributions (%) for the europium compounds based on phenyl-containing CAPhs at 298 K.

Coordination compound	Photoluminescence intensity distribution (%)					Red/orange ratio
	^5^D_0_→^7^F_0_	^5^D_0_→^7^F_1_	^5^D_0_→^7^F_2_	^5^D_0_→^7^F_3_	^5^D_0_→^7^F_4_	
Cs[EuL^1^_4_]	0.6	17.8	50.3	3.7	27.6	2.9
NEt_4_[EuL^1^_4_]	0.1	17.6	46.4	2.8	33.1	2.6
[EuL^1^_3_phen]	0.3	12.7	62.4	1.6	23.1	4.6
[EuL^1^_3_bipy]	0.1	12.0	62.1	2.5	23.2	4.4
[EuL^1^_3_TPPO]	0.4	8.9	76.5	3.6	10.5	9.9
[Eu(HL^2^)_3_(NO_3_)_3_]	0.4	13.9	62.8	2.7	20.2	4.7
[EuL^2^_3_phen]	0.2	11.1	63.2	3.6	21.8	5.7
[EuL^2^_3_bipy]	0.5	11.0	63.1	4.0	21.5	5.7
NEt_4_[EuL^3^_4_]	0.3	13.3	46.8	4.4	35.1	3.9
PPh_4_[EuL^3^_4_]	0.6	7.8	53.4	4.7	33.6	8.2
[EuL^3^_3_phen]	0.2	8.6	53.4	3.6	34.3	6.2
[EuL^3^_3_bipy]	0.2	14.3	62.2	2.0	21.3	4.4

The value of the red/orange ratio for Eu^3+^ compounds with CAPhs ligand (from 2.6 to 9.9) is smaller than for Eu^3+^ β-diketonates (from 7 to 27) (Klink et al., 2000; Binnemans et al., 2005). Unfortunately, based on these values it is not possible to draw conclusions about the comparison of the symmetry of the surroundings of the Eu^3+^ ion (Tanner, 2013; Binnemans, 2015) in compounds with CAPhs ligands and β-diketones. It should be remembered that the red/orange ratio is affected by various factors, including the polarizability of the ligands.

The quite high intensity of the ^5^D_0_→^7^F_4_ transition bands for tetrakis-compounds should be noted compared to tris-compounds with different ligands (Kariaka et al., 2022). Such an unusual behavior was earlier observed for some compounds and explained by the deviation of the EuO8 coordination polyhedron from the cubic geometry to D_2_ (Bettinelli et al., 2011) or by the highly polarized environment of Eu^3+^ with local symmetry corresponding to the D_4d_ coordination geometry (Sá Ferreira et al., 2006).

The decay curves for all the considered CAPh-based europium coordination compounds have a monoexponential character. The luminescence lifetime does not depend on temperature (Table 6), which proves the absence of temperature-dependent non-radiative transitions in the compounds. Cs[EuL^1^_4_] shows the highest value of the luminescence lifetime. The lowest luminescence lifetime is observed for the mixed-ligand compounds [EuL^2^_3_phen], [EuL^2^_3_bipy], and the tetrakis-compound PPh_4_[EuL^3^_4_]. The ^5^D_0_ emission lifetime is dependent on the excitation wavelength, which is characteristic of many Eu^3+^ compounds with different ligands (Ferreira et al., 2012). This phenomenon is explained by the fact that the lifetime is related to the process of changing the occupancy levels of the lanthanide over time, and the latter depends on the presence or absence of energy transfer channels in the lanthanide itself and in its surroundings.

**TABLE 6:** Emission decay times of the Eu^3+^ coordination compounds at different wavelength excitations.

Coordination compound	τ, ms λ_exc._ = 254 nm at 298 K	τ, ms λ_exc._ = 280 nm		τ, ms λ_exc._ = 320 nm (for [EuL_3_phen]) λ_exc._ = 350 nm (for [EuL_3_bipy])	
		298 K	77 K	298 K	77 K
Cs[EuL^1^_4_]	3.5	2.7	2.6	—	—
NEt_4_[EuL^1^_4_]	2.2	—	—	—	—
[EuL^1^_3_phen]	1.8	—	—	1.6	1.5
[EuL^1^_3_bipy]	1.9	—	—	1.7	1.8
[EuL^1^_3_TPPO]	—	1.3	1.2	—	—
[Eu(HL^2^)_3_(NO_3_)_3_]	1.9	1.8	2.0	—	—
[EuL^2^_3_phen]	1.5	1.3	1.4	1.2	1.3
[EuL^2^_3_bipy]	1.4	1.3	—	1.2	—
NEt_4_[EuL^3^_4_]	1.8	1.7	1.8	—	—
PPh_4_[EuL^3^_4_]	1.4	1.5	1.6	—	—
[EuL^3^_3_phen]	1.9	1.7	1.7	1.6	1.6
[EuL^3^_3_bipy]	2.0	1.7	1.8	1.6	1.4

— Not measured.

**FIGURE 7:**
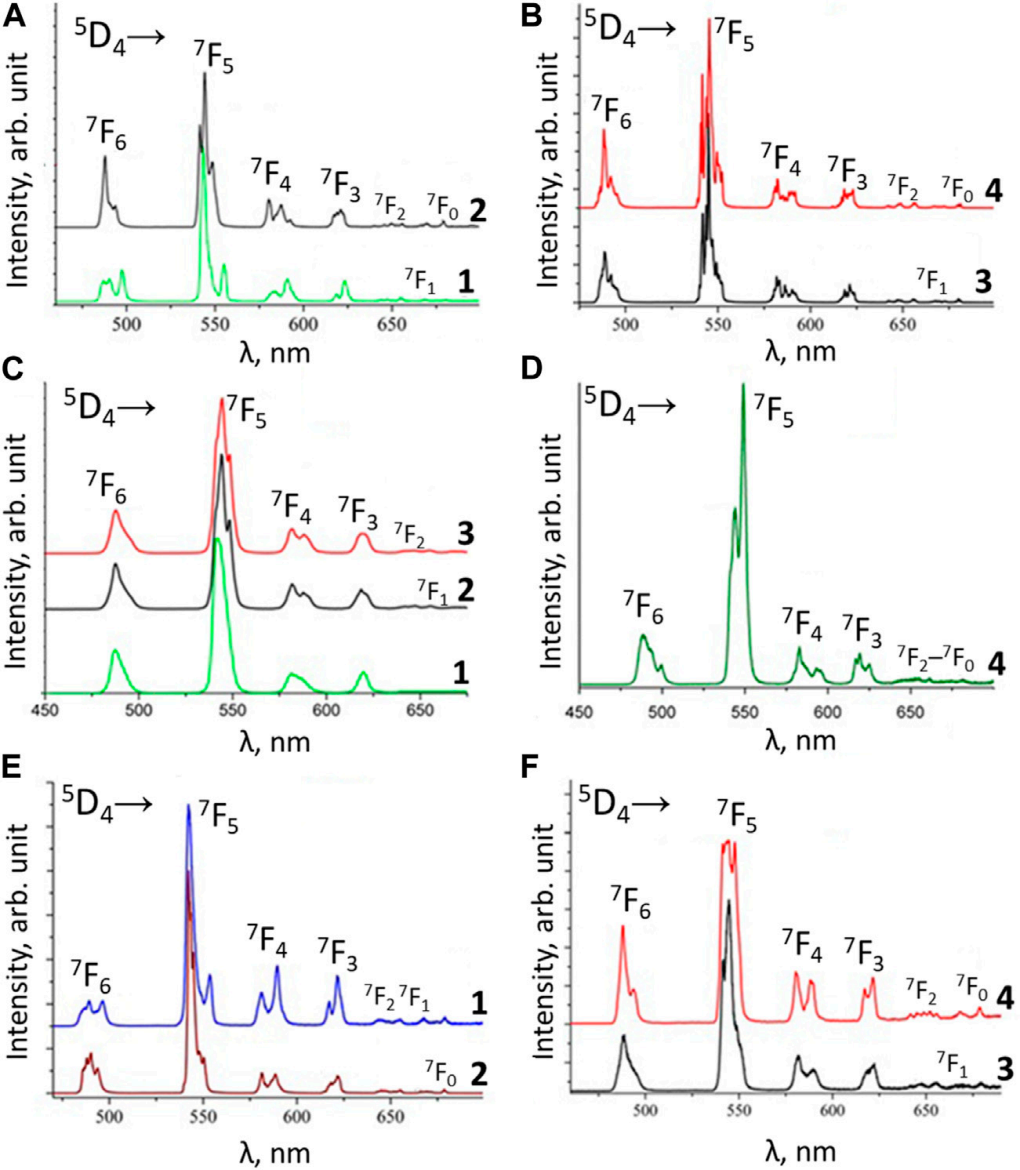
Low temperature (77 K) emission spectra of the terbium complexes: **(A)** NEt_4_[TbL^1^_4_] (1) and Cs[TbL^1^_4_] (2); **(B)**- [TbL^1^_3_Phen] (3) and [TbL^1^_3_bipy] (4); **(C)** [Tb(HL^2^)_3_(NO_3_)_3_] (1), [TbL^2^_3_Phen] (2) and [TbL^2^_3_bipy] (3); **(D)** [TbL^1^_3_TPPO] (4); **(E)** NEt_4_[TbL^3^_4_] (1) and PPh_4_ [TbL^3^_4_] (2); **(F)** [TbL^3^_3_Phen] (3) and [TbL^3^_3_bipy] (4).

The luminescence spectra of the Tb^3+^ coordination compounds [TbL_3_bipy], [TbL_3_phen] (L = L^1^, L^2^, L^3^), Cat[TbL_4_] (L = L^1^, L^3^), [TbL^1^_3_TPPO], and [Tb(HL^2^)_3_(NO_3_)_3_] are shown in Figure 7.

Similar to the Eu^3+^ coordination compounds, the luminescence spectra of the Tb^3+^ coordination compounds, obtained upon excitation into the ligand absorption bands, are characterized by the presence of narrow bands, corresponding to Tb^3+^ 4f-4f transitions ^5^D_4_→^7^F_J_ (J = 6–0) and absence of the ligand’s fluorescence band. This points to an effective excitation energy transfer from the ligands to the Eu^3+^ and Tb^3+^ ions. An effective excitation energy transfer is also confirmed by the excitation spectra, in which the intensities of the ligands’ absorption bands are significantly higher compared to f-f transitions of the Eu^3+^ and Tb^3+^ ions. The bands with maxima at ∼490, ∼545, ∼680, ∼620, ∼650, ∼670, and ∼680 nm in the luminescence spectra of the Tb^3+^ coordination compounds are assigned to ^5^D_4_-^7^F_J_ (J = 6–0) transitions, respectively. The band of ^5^D_4_→^7^F_5_ transition dominates in intensity in all the spectra. It is known that the luminescence spectra of terbium have a more complex Stark structure compared to the spectra of europium. In addition, in the case of terbium, even at 77 K, the Stark sublevels of the term ^5^D_4_ remain populated. These factors make it difficult to analyze the environment around the Tb^3+^ ion in compounds according to spectral criteria.

**FIGURE 8:**
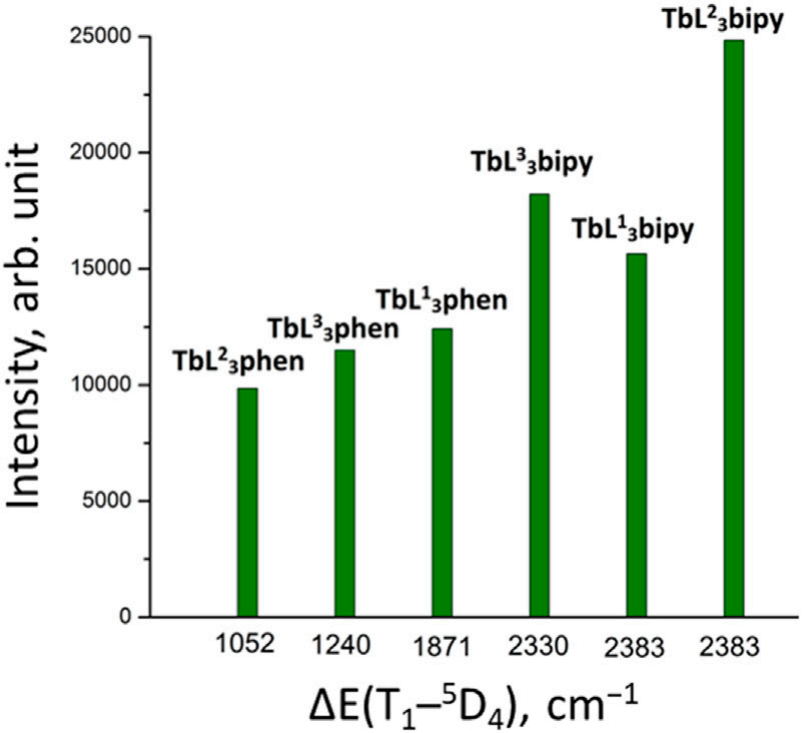
The luminescence intensity of [TbL_3_phen] and [TbL_3_bipy] coordination compounds for solid samples at room temperature.

The Tb^3+^ luminescence intensity was measured for powdered samples. The coordination compounds’ crystals were ground thoroughly with a mortar and pestle for 2-3 min. The obtained powders were loaded in a solid sample cuvette (cell) supplied with Fluorolog FL 3–22. The front surface of each sample was also examined to ensure the absence of cracks and crevices in the powder. Such cracks and crevices were removed by gently tapping the cuvette or by gently pressing on the sample with a spatula and tracing paper. The [TbL^2^_3_bipy] exhibited the highest emission intensity among the examined Tb^3+^ coordination compounds (Table 7). The lowest luminescence intensity was found for [Tb(HL^2^)_3_(NO_3_)_3_]. It should be noted that, in contrast to europium compounds, the terbium mixed ligand tris-compounds with 1,10-phenanthroline show lower emission intensity compared to coordination compounds with 2,2′-bipyridyne, which can be explained by better matching of the triplet level of 2,2′-bipyridyne with terbium ^5^D_4_ level, compared to 1,10-phenathroline. The [TbL_3_phen] luminescence intensity follows the trend of the ligands’ triplet state energy reduction (Figure 8). However, different emission intensities are observed for [TbL_3_bipy] compounds at the same ΔE value, which shows the importance of other factors in the sensitization process as well.

**TABLE 7:** Experimental luminescence characteristics of some phenyl-containing CAPh-based Tb^3+^ coordination compounds in crystal state at room temperature.

Coordination compound	Intensity (λ_ex_ = 270 nm)	τ, ms		λ_ex_, nm
		298 K	77 K	
[Tb(Wo)_3_Q]	—	1.7	—	280
Cs[TbL^1^_4_]	1.5	2.8	2.6	273
NEt_4_[TbL^1^_4_]	1.4	2.4	—	270
[TbL^1^_3_phen]	2.0	1.6	1.6	345
[TbL^1^_3_bipy]	2.6	1.8	1.8	330
TbL^1^_3_TPPO	—	1.6	1.6	285
[Tb(HL^2^)_3_(NO_3_)_3_]	1	1.7	—	270
[TbL^2^_3_phen]	1.7	1.6	1.7	346
		1.8	1.9	280
[TbL^2^_3_bipy]	4.0	1.4	—	320
		1.5		276
[TbL^3^_3_phen]	1.9	1.6	1.6	343
		1.6	1.7	273
[TbL^3^_3_bipy]	3.0	1.9	1.9	323
		2.0	2.0	273
NEt_4_[TbL^3^_4_]	2.7	2.0	2.2	273
PPh_4_[TbL^3^_4_]	2.8	1.3	1.3	300

## 8 Conclusions and perspectives

This review summarizes studies on luminescent lanthanide coordination compounds based on carbacylamidophosphates and focuses on the thermal behavior and luminescence of Eu^3+^ and Tb^3+^ coordination compounds. The nature of lanthanides luminescence and ways of its enhancement are described.

Comparing CAPh-based compounds to commonly used phosphors like β-diketonates can help showcase the distinctive thermal and spectral properties of CAPh-based compounds. Additionally, theoretical methods have proven to be effective in quantifying the energy transfer process between these classes of ligands and the Ln^3+^ ion.

Being close structural analogues of β-diketones, carbacylamidophosphates, however, differ from the former in their coordination behavior, spectral properties, and resistance to exposure to UV radiation, and thus are considered a promising new class of ligands for the design of highly luminescent materials for practical use. Phenyl-containing CAPh-based Eu^3+^ and Tb^3+^ coordination compounds possess intense, long-lasting luminescence. The rather high energy of the triplet state of the carbacylamidophosphates and the suppression of multiphonon emission quenching provides possibilities to use them for sensitization of a wider list of lanthanides, emitting in the visible region, including Dy^3+^ yellow/white emission and Tm^3+^ blue emission as well as in the NIR range - Nd^3+^, Er^3+^, and Yb^3+^.
